# Identification of kidney stones in KUB X-ray images using VGG16 empowered with explainable artificial intelligence

**DOI:** 10.1038/s41598-024-56478-4

**Published:** 2024-03-14

**Authors:** Fahad Ahmed, Sagheer Abbas, Atifa Athar, Tariq Shahzad, Wasim Ahmad Khan, Meshal Alharbi, Muhammad Adnan Khan, Arfan Ahmed

**Affiliations:** 1 School of Computer Science, National College of Business Administration and Economics, Lahore, 54000 Pakistan; 2 Department of Computer Sciences, Bahria University, Lahore Campus, Lahore, 54000 Pakistan; 3 Department of Computer Science, Comsats University Islamabad, Lahore Campus, Lahore, 54000 Pakistan; 4 Department of Computer Sciences, COMSATS University Islamabad, Sahiwal Campus, Sahiwal, 57000 Pakistan; 5 Department of Computer Science, College of Computer Engineering and Sciences, Prince Sattam Bin Abdulaziz University, 11942 Alkharj, Saudi Arabia; 6 School of Computing, Skyline University College, University City Sharjah, 1797 Sharjah, UAE; 7 Department of Software, Faculty of Artificial Intelligence and Software, Gachon University, Seongnam-si, 13120 Republic of Korea; 8 Riphah School of Computing and Innovation, Faculty of Computing, Riphah International University, Lahore Campus, Lahore, 54000 Pakistan; 9 AI Center for Precision Health, Weill Cornell Medicine-Qatar, Doha, Qatar

**Keywords:** Artificial intelligence (AI), Machine learning (ML), Deep learning (DL), Convolutional neural network (CNN), Transfer learning (TL), VGG16, Kidney-ureter-bladder (KUB), Kidney stones, Explainable artificial intelligence (XAI), Layer-wise relevance propagation (LRP), Kidney diseases, Computer science

## Abstract

A kidney stone is a solid formation that can lead to kidney failure, severe pain, and reduced quality of life from urinary system blockages. While medical experts can interpret kidney-ureter-bladder (KUB) X-ray images, specific images pose challenges for human detection, requiring significant analysis time. Consequently, developing a detection system becomes crucial for accurately classifying KUB X-ray images. This article applies a transfer learning (TL) model with a pre-trained VGG16 empowered with explainable artificial intelligence (XAI) to establish a system that takes KUB X-ray images and accurately categorizes them as kidney stones or normal cases. The findings demonstrate that the model achieves a testing accuracy of 97.41% in identifying kidney stones or normal KUB X-rays in the dataset used. VGG16 model delivers highly accurate predictions but lacks fairness and explainability in their decision-making process. This study incorporates the Layer-Wise Relevance Propagation (LRP) technique, an explainable artificial intelligence (XAI) technique, to enhance the transparency and effectiveness of the model to address this concern. The XAI technique, specifically LRP, increases the model's fairness and transparency, facilitating human comprehension of the predictions. Consequently, XAI can play an important role in assisting doctors with the accurate identification of kidney stones, thereby facilitating the execution of effective treatment strategies.

## Introduction

Urolithiasis, or kidney stones, is one of the most common urological conditions worldwide^1^. Kidney stones are hard concretion or stone-like pieces that form in the kidneys due to dietary minerals in the urine^2^. Symptoms, including flank pain, nausea, and vomiting, can indicate kidney stones^3^. While they can manifest in individuals of any gender, prevalence is higher in males, with approximately 7% of females and 13% of males experiencing them in their lifetime^4^. Factors such as dietary habits, sedentary lifestyle, diabetes mellitus, obesity, hypertension, and metabolic syndrome elevate the risk of stone formation^5^.

Medical professionals use imaging techniques to identify kidney stones removed by surgical intervention. After treatment, kidney stones may recur and develop into a chronic condition after treatment and kidney malfunctions can be life-threatening^6^. The ureter can become blocked depending on the size of the stone, causing significant pain, particularly in the lower back, although it can hurt the groin^7^. Older people are more likely to report atypical or no pain when passing a stone, making diagnosing kidney stone disease challenging in this demographic^8^. Different stages of disease evaluation employ various imaging techniques. The typical imaging techniques for examining kidney stones are sonography^9^, computed tomography (CT)^10^, and KUB X-ray imaging^11^. Sonography, also known as ultrasonography or simply ultrasound, is a quick, safe, and easy procedure that can provide valuable evidence for a kidney stone diagnosis. Still, its sensitivity for detecting kidney stones is limited. CT can identify kidney stones and determine their number, location, and size; however, it involves exposure to ionizing radiation. KUB X-ray can also detect kidney stones and provide essential information regarding their classification, shape, number, position, and size. In this context, the most popular method is two plain KUB X-ray imaging, which is already available, less expensive, and exposes patients to less radiation than CT^12^.

**Figure 1:**
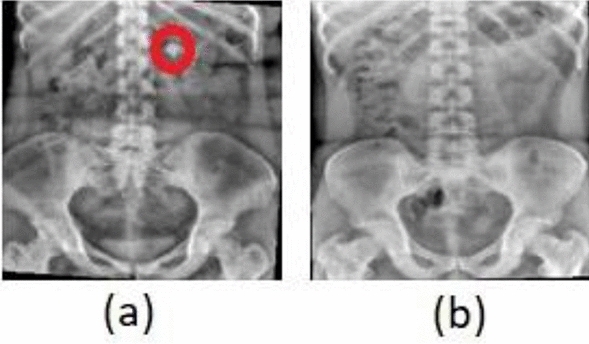
KUB x-ray sample images: (**a**) Kidney Stone; (**b**) Normal.

One of the most crucial stages in locating, measuring, and identifying the composition of kidney stones before and during treatment involves using KUB x-ray imaging, which is also employed to evaluate prognosis. Figure 1 displays samples of KUB X-ray images depicting kidney stones and normal images used in this article.

Nephrologists typically use KUB X-ray images to identify kidney stones. This information helps determine whether the individual is healthy or a patient requiring treatment. Directing an X-ray beam through the body obtains a KUB radiograph. The resulting image appears in shades of black and white, depending on the varying densities and X-ray absorption of different body parts. Muscles and fat appear grey due to medium densities, while bones appear white due to their high density. The low density of the air in the lungs makes them appear black on the radiograph.

Given the increasing prevalence and the complexity of diagnosing kidney stones, there is an urgent need for innovative diagnostic techniques. Traditional imaging techniques may produce low-quality images, making interpreting results easier. This limitation has led to the exploration of new methods, including using artificial intelligence (AI) to improve diagnostic accuracy. Integrating AI into novel diagnostic methodologies holds significant promise for refining diagnostic accuracy and facilitating therapeutic interventions^13^.

Machine learning (ML), a subfield of AI, is widely considered a powerful tool for enhancing disease prediction and diagnosis^14,15^. Recently, there has been a substantial increase in the quantity and quality of research focused on utilizing ML for automatic disease identification. However, effective feature extraction methods are essential for improving ML models. The need for the manual formulation of complex hypotheses in traditional ML classifiers constitutes a disadvantage^16^. In contrast, deep neural networks (DNNs) can autonomously generate complex hypotheses, rendering them practical for learning nonlinear correlations^16^. This autonomous capability is part of why deep learning (DL), a subset of which includes DNNs, has historically diverged from traditional ML methods^17^. Due to their enhanced efficacy in processing large-scale data sets, their ability to extract hidden valuable knowledge from data, and to employ specific pre-trained networks, DL models are therefore frequently used in medical imaging systems.

DL can learn from and model vast amounts of data^18^. Due to their advanced information processing capabilities, DL models can effectively represent complex, high-dimensional datasets^19^. Deep models have been effectively applied in various applications, including lesion detection^20,21^, classification^22–25^, object tracking^26^, image super-resolution reconstruction^27^, image inpainting^28–32^, and segmentation of medical images^33,34^. Autoencoder (AE), Recurrent Neural Networks (RNN), Deep Belief Networks (DBN), Direct Deep Reinforcement Learning, Recursive Neural Networks, and Convolutional Neural Networks (CNN) are standard DL techniques^35^. CNNs are frequently used in DL to automatically learn features, which are then used for classification and detection^36,37^.

DL approaches find extensive use within the healthcare sector. However, these models, often called “black boxes,” challenge our understanding of the rationale behind their decisions or predictions, and the absence of interpretability can create issues. In this context, implementing explainable AI (XAI) techniques can enhance transparency and improve understanding of its decisions. This article introduces a model that suggests identifying kidney stones by applying transfer learning (TL) empowered by XAI.

The development of medical image processing methods has accelerated the introduction of smart prediction and diagnosis tools^38^. AI can assist doctors in making better clinical decisions in specific functional areas of healthcare, such as radiography, or may even replace human judgment in certain circumstances^39^. AI employs DL, ML, and other learning-based methods^40^. Recent research has shown the utilization of DL techniques in specific applications, including an enhanced rime optimization-driven multi-threshold segmentation for COVID-19 X-ray images^41^, high-precision multiclass classification of lung diseases using customized MobileNetV2^42^, phase retrieval for X-ray differential phase contrast radiography with knowledge transfer learning^43^, attention-based VGG-16 model for COVID-19 chest X-ray image classification^44^, and pre-trained VGG-16 with CNN architecture for classifying X-ray images into normal or pneumonia categories^45^. Researchers in the field of AI have created numerous ML and DL algorithms for detecting kidney stones over the past few decades.

For the Computer-aided diagnosis (CAD) of kidney stones, Ishioka et al.^46^ employed a CNN (ResNet) method utilizing over 1000 KUB x-ray images from three hospitals. The researchers used 190 as testing data and 827 as training data. The test dataset’s precision, sensitivity, and F1 score were 0.49, 0.72, and 0.58, respectively. Chiang et al.^47^ introduced an algorithm for detecting kidney stones using an artificial neural network (ANN) and discriminant analysis (DA) in conjunction with genetic polymorphisms and environmental factors such as milk consumption, water consumption, and outdoor activities. The research revealed that considering only genetic factors does not produce noticeable distinctions in the success of the models. However, considering the environmental and genetic factors, the ANN model outperforms the DA model with 89% accuracy.

Dussol et al.^48^ implemented ANN models to examine 11 clinical and biochemical markers in 119 males with kidney stone formation and 96 males in the control group. Using linear discriminant analysis (LDA), they accurately identified 75.8% of the cases. Multivariate discriminant analysis (MVDA) accurately classified 74.4% of the patients.

In a parallel investigation, Cauderella et al.^49^ implemented the ANN models in conjunction with traditional statistical methodologies to predict the recurrence of incidents within a five-year timeframe post-initial clinical diagnosis and metabolic assessment. They based their model on a dataset from 80 patients with kidney stone disease. Owing to its established reliability as a traditional statistical technique, logistic regression (LR) was selected as a comparison tool for ANN. The same training and testing sets as for ANN were used to create and test LR. The statistical software Statistical Package for the Social Sciences (SPSS) was used to develop LR. The ANN model demonstrated a predictive accuracy of 88.8%, significantly outperforming the LR model, which yielded an accuracy rate of 67.5%.

In a separate investigation conducted by Kumar and Abhishek^50^, researchers made a comparative analysis to evaluate the diagnostic efficacy of three distinct neural network algorithms: Learning Vector Quantization (LVQ), Multilayer Perceptron (MLP), and Radial Basis Function (RBF). They compared the algorithms in terms of their level of accuracy, training dataset size, and the time required to construct a model. The MLP algorithm emerged as the most productive, with an accuracy of 92%, thereby establishing itself as an optimal tool for the early detection of kidney stones in patients and reducing the time required for diagnosis.

Ebrahimi and Mariano^51^ created a semi-automated program to enhance kidney stone detection in KUB computed tomography (KUB CT) analysis using image processing techniques and geometry principles. The program outlines and segments the kidney area, identifies kidney stones, and determines their size and position using pixel count metrics. An evaluation of the framework's performance on KUB CT scans from a cohort of 39 patients yielded a detection accuracy of 84.61%, indicating its potential to augment diagnostic precision in kidney stone identification. Kazemi and Mirroshandel^52^ proposed a novel method for predicting the chance of a kidney stone using ensemble learning. They sourced data from 936 patients diagnosed with nephrolithiasis at the Renal Center of the Razi Hospital in Rasht between 2012 and 2016. The ensemble-based model's final accuracy was 97.1%. Li and Elliot^53^ conducted a study to assess the accuracy of natural language processing (NLP) in recognizing a group of patients (n = 1874) with positive CT KUB results for renal stones. The NLP achieved an accuracy rate of 85%.

De Perrot et al.^54^ developed an ML algorithm that employs radiomics feature extraction from low-dose CT (LDCT) images to differentiate between kidney stones and phleboliths. This ML classification model, trained on radiomics characteristics, achieved an overall accuracy of 85.1% on the independent testing set. In another study, Kahani et al.^55^ presented a classification technique for urinary stones utilizing KUB x-ray images. They employed the least absolute shrinkage and selection operator (LASSO) algorithm with ML classifiers. This methodology yielded a classification accuracy of 96% for kidney stones. Jungmann et al.^56^ created an NLP technique trained on subjective assessment to automatically collect positive hit rates and clinical information to evaluate 1714 narrative LDCT reports. In 38% of occurrences, there was a minimum of one kidney stone, and in 45%, there was a minimum of one ureter stone.

Annameti Rohith et al.^57^ developed a technique employing median and rank filters to increase the detection rate of identifying kidney stones in ultrasound images regarding accuracy and sensitivity. They evaluated the median and rank filters for their accuracies and sensitivities using a MATLAB simulation tool with a sample size 114 and a *p* value of 0.8. The median filter achieved an accuracy of 86.4%, the rank filter attained an accuracy of 82.2%, the median filter's sensitivity was 87.7%, and the rank filter's sensitivity was 82.5%. The median filter significantly outperformed the rank filter in both accuracy and sensitivity. Suresh and Abhishek^58^ proposed image-processing techniques to detect kidney stones in KUB ultrasound images, including pre-processing, segmentation, and morphology. Their model achieved an accuracy of 92.57% in kidney stone detection.

To discriminate between distal ureteric calculi and phleboliths using the characteristics of non-contrast CT (NCCT) images, Jendenber et al.^59^ trained and created a CNN model. They then compared their findings to the assessments of seven professional radiologists. The radiologists' accuracy was 86%, whereas the CNN model's was significantly higher at 92%. Cui et al.^60^ proposed a DL and threshold-based model for detecting kidney stones. They performed experiments employing a small dataset of 625 CT images and achieved an accuracy of 90.30% and a sensitivity of 95.9%.

Yildirim et al.^61^ proposed a DL model for automated kidney detection utilizing 1799 coronal CT images. For kidney stone detection, they used XResNet-50. Using CT images to identify kidney stones, the designed automated model obtained a 96.82% identification rate. Tsitsiflis et al.^62^ constructed an ANN to evaluate extracorporeal shockwave lithotripsy (ESWL) parameters in patients with urinary lithiasis. Medical data from 716 patients were collected. 549 were used for training, 167 for testing, and 12 nodes were used as inputs for the ANN. The ANN achieved a testing accuracy of 81.43%.

Valencia et al.^63^ introduced an image-processing methodology for detecting kidney stones in CT scans. The study comprised four steps: image preprocessing with a median filter, segmentation using the k-means clustering algorithm, kidney stone detection, and classification. The team gathered data from approximately 40 patients diagnosed with kidney stone diseases, utilizing CT scans in a clinical setting. The novel approach in this study aimed to detect boundaries and segment areas and enhance kidney stone detection through pixel-level analysis. This methodology enables both the localization of kidney stones and the quantification of affected patients. The algorithm achieved an accuracy rate of 92.5%.

While existing literature has made valuable contributions to the field, some areas could benefit from further exploration (Table 1 outlines gaps identified in previous research). Given the identified research gaps, our proposed method aims to overcome these limitations and drive progress in kidney stone identification. The main motivations and innovations of our work are outlined below:

1. The studies encompassed in the review, ranging from references^47–63^, have not incorporated data augmentation methodologies. Data augmentation methodologies improve model performance, reduce overfitting, and enhance the ability of the model to generalize to new, unseen data.
2. Previous literature^47–63^ may have yet to attain optimal accuracy in identifying and predicting kidney stones. Improved accuracy increases the chances of identifying kidney stones.
3. Current models could benefit from enhanced transparency and fairness to improve the interpretability of their predictions. A deeper understanding of the decision-making process and contributing factors is essential for achieving more transparent, fair, and effective diagnostic outcomes.

**Table 1:** Limitations and outcomes of previous work.

Author	Dataset	Dataset type	Technique	Outcomes	Limitations		
					Data augmentation	Improvable Accuracy	Use of XAI
Chiang et al.^47^	105 healthy controls and 151 patients with calcium oxalate stones	Handcrafted features	ANN and DA	ANN achieved an accuracy of 89%, while DA achieved 75%	No	Yes	No
Dussol et al.^48^	119 stone formers and 96 controls	Handcrafted features	ANN (LDA and MVDA)	ANN with LDA gives a high accuracy of 75.8% compared to ANN with MVDA (74.4%)	No	Yes	No
Cauderella et al.^49^	80 patient’s data	Handcrafted features	ANN and LR	Comparing the performance of ANN with LR and then observing that ANN gives better accuracy of 88.8% compared to LR (67.5%)	No	Yes	No
Kumar and Abhishek^50^	Data from 1000 patients	Handcrafted features	LVQ, MLP, and RBF	The accuracy obtained by LVQ, MLP, and RBF is 84%, 92%, and 87%, respectively	No	Yes	No
Ebrahimi and Mariano^51^	KUB CT scan slides from 39 patients	Image-based	Image processing techniques and geometry principles	Detect kidney stones with an accuracy of 84.61%	No	Yes	No
Kazemi and Mirroshandel^52^	Numeric characteristics from 936 patients	Handcrafted features	Ensemble learning model	Obtained an accuracy of 97.1%	No	Yes	No
Li and Elliot^53^	1874 CT KUB reports	Handcrafted features	NLP	An overall accuracy of 85% was attained by applying NLP to CT KUB reports	No	Yes	No
De Perrot et al.^54^	416 patient data	Handcrafted features	ML model	Using a machine learning model results in an overall accuracy of 85.1%	No	Yes	No
Kahani et al.^55^	KUB x-ray images	Image-based	LASSO with ML classifiers	Obtained an accuracy of 96%	No	Yes	No
Jungmann et al.^56^	1714 LDCT images	Image-based	NLP	Applying NLP to 1714 LCDT images achieves an overall accuracy of 72%	No	Yes	No
Annameti Rohith et al.^57^	114 ultrasound images	Image-based	Median and rank filters	When applied to 114 ultrasound images, the median filter gives an overall high accuracy of 86.4% compared to the rank filter (82.2%)	No	Yes	No
Suresh and Abhishek^58^	KUB ultrasound images	Image-based	Image processing techniques	The proposed model gives an accuracy of 92.57% for stone detection	No	Yes	No
Jendenber et al.^59^	NCCT images of 341 patients containing a distal ureteral stone, phlebolith, or both	Image-based	CNN	CNN differentiated stones and phlebolith with 92% accuracy	No	Yes	No
Cui et al.^60^	625 CT images	Image-based	DL and threshold-based model	Achieved an accuracy of 90.30%	No	Yes	No
Yildirim et al.^61^	1799 coronal CT images	Image-based	XResNet-50	Using CT images, XResNet-50 demonstrated an accuracy of 96.82%	No	Yes	No
Tsitsiflis et al.^62^	Medical data of 716 patients	Feature-based data	ANN	Achieved a testing accuracy of 81.43%	No	Yes	No
Valencia et al.^63^	CT scans of around 40 patients	Image-based	Image processing	Achieved an accuracy of 92.5% for stone detection	No	Yes	No

For this paper, the main contributions are as follows:

1. The proposed research introduces a novel deep TL model that autonomously extracts relevant features from KUB X-ray images. This model successfully identifies the presence of kidney stones in these images.
2. The proposed model uses various performance measures, including accuracy, misclassification rate, precision, sensitivity, specificity, false positive rate (FPR), false negative rate (FNR), and F1 Score. The evaluations show that the model performs reliably and commendably.
3. The study conducts a comparative analysis between the proposed model and existing methodologies documented in the literature^47–63^. This evaluation reveals that the proposed model achieves higher accuracy than previous approaches, thus showcasing its superiority in kidney stone identification.
4. The research includes a technique called XAI, specifically layer-wise relevance propagation (LRP), to improve the transparency and fairness of the model's predictions. LRP helps clarify the reasoning behind the model's predictions, thereby promoting transparency and fairness in the kidney stone identification process.

The rest of the article is divided into the following sections: The proposed model's methodology is described in Section “Methodology”. Simulation and results are presented in Section “Simulation and results”. The conclusion is presented in Section “Conclusion”. Limitations and future work are briefly discussed in Section “Limitations and future work”.

## Methodology

**Figure 2:**
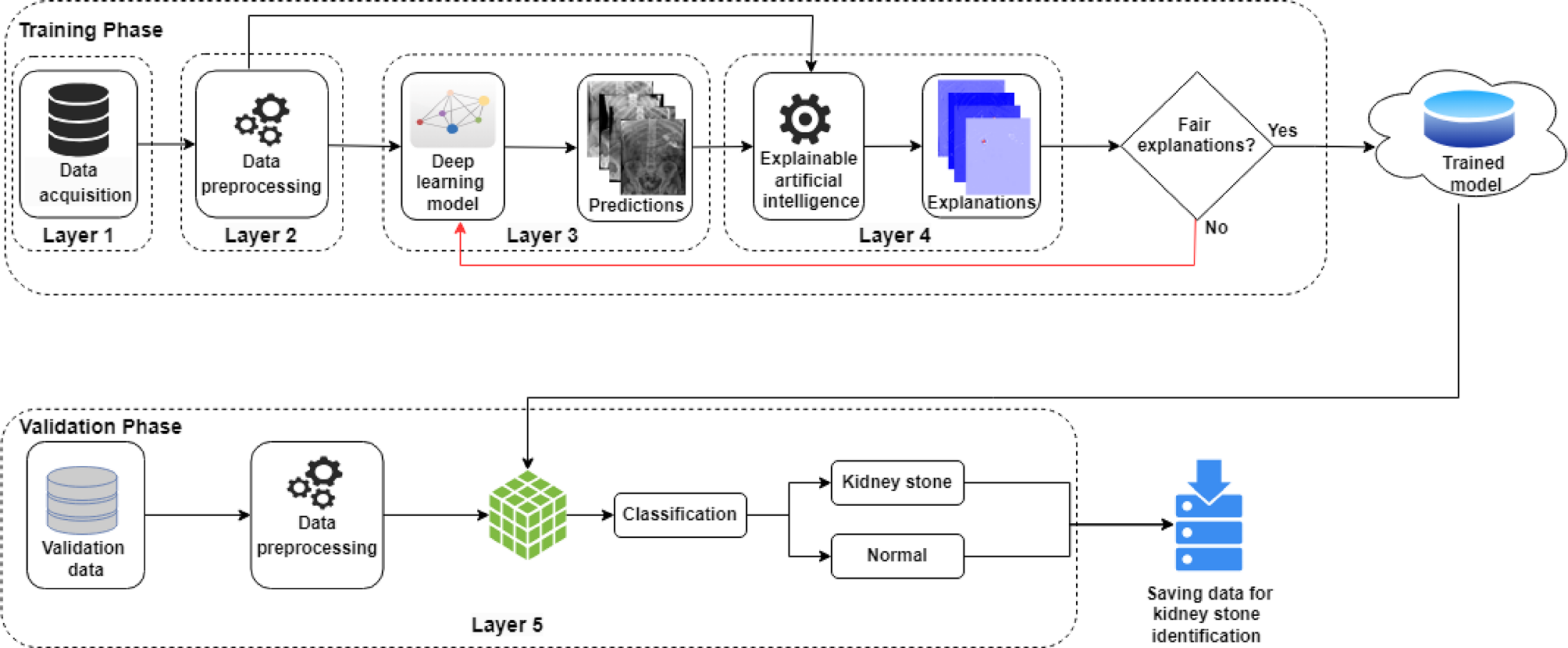
Proposed kidney stone identification model using DL empowered with XAI.

The proposed kidney stone identification model employs DL empowered with XAI (Fig. 2). The model consists of five layers and two phases: training and validation. During the training phase, Layer 1 is dedicated to acquiring raw kidney-ureter-bladder (KUB) x-ray images, categorized as either 'kidney stone' or 'normal.' These images are high-resolution JPEG files with dimensions exceeding 2000 × 2000 pixels. In Layer 2, raw data undergoes preprocessing per the requirements of the DL model. The images are resized to dimensions of 224 × 224 × 3 and converted into PNG format. In this context, '224 × 224' signifies length and width, and '3' denotes the number of channels. Following preprocessing, data is separated between training and testing, with 70% allocated for training and 30% for testing. The pre-trained VGG16 model is imported and customized to the DL model.

Layer 3 describes the predictions made by the DL model. While these predictions hold potential utility in decision-making, they do not offer insights into the model's reasoning, thus making it a 'black box.' To mitigate this, Layer 4 incorporates XAI into the model. This feature compares the DL model's predictions with the preprocessed data to furnish explanations. If the explanations are unfair, the model is retrained; otherwise, it is stored on the cloud.

Layer 5 represents the validation phase of the model, wherein the trained model is imported from the cloud to validate the pre-processed data acquired from various sources. The proposed model intelligently classifies the KUB x-ray images into two classes with explanations. Following the successful identification of kidney stones, the system saves the corresponding data.

Table 2 represents the pseudocode for the proposed kidney stone identification model.

**Table 2:**
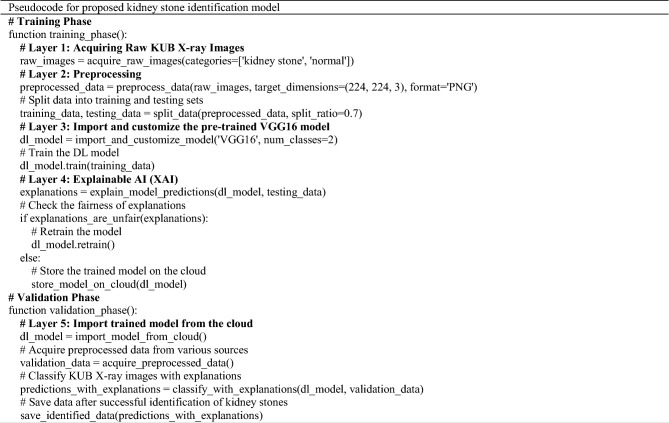
Pseudocode for proposed kidney stone identification model.

### KUB x-ray images dataset

KUB X-ray images were acquired from the Department of Urology and Kidney Transplantation at MAYO Hospital in Lahore, Pakistan. The dataset consists of 500 KUB X-ray images selected from patients who had undergone radiographic examinations for kidney stones between February 2021 and October 2022. The images were obtained through the anteroposterior (AP) view. Two radiology specialists examined the collected KUB X-ray images and determined the presence or absence of kidney stones. Of the 500 images, 250 were identified as exhibiting kidney stones, while the remaining 250 were not. Subsequently, the images were augmented into 14,265 KUB x-ray images. Within this augmented dataset, 8941 images displayed instances of kidney stones, while 5324 images represented normal cases. As mentioned in the methodology section, the dataset is divided into 70:30. The number of training images is 9986 (kidney stone 6259, normal 3727), while the number of testing images is 4279 (kidney stone 2682, normal 1597).

### TL

TL is a technique for applying a model's previously acquired knowledge to a new dataset^64^. TL enables the utilization of highly competent, pre-trained networks rather than creating CNNs for each application. The core idea is that specific applications can be modeled by training a large model on a diverse and broad dataset. The initial layers will learn generic properties such as color, while later layers will serve particular applications. A pre-trained model, VGG16, is employed in this article to identify and predict kidney stones.

#### VGG16

**Figure 3:**
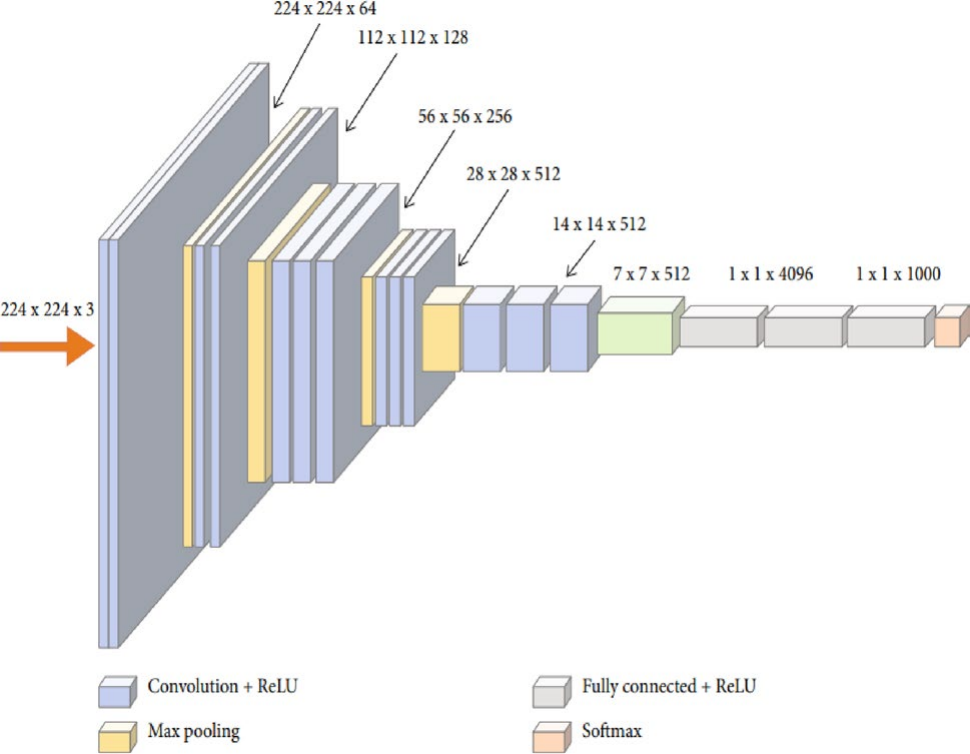
VGG16 original architecture^66^.

In 2014, Simonyan and Zisserman introduced VGG16, a TL-based CNN model characterized by a sequential network structure^65^. VGG16 is a deep CNN architecture with a total of 16 layers^65,66^, which includes 13 convolutional layers and 3 fully connected dense layers (Fig. 3).

**Figure 4:**
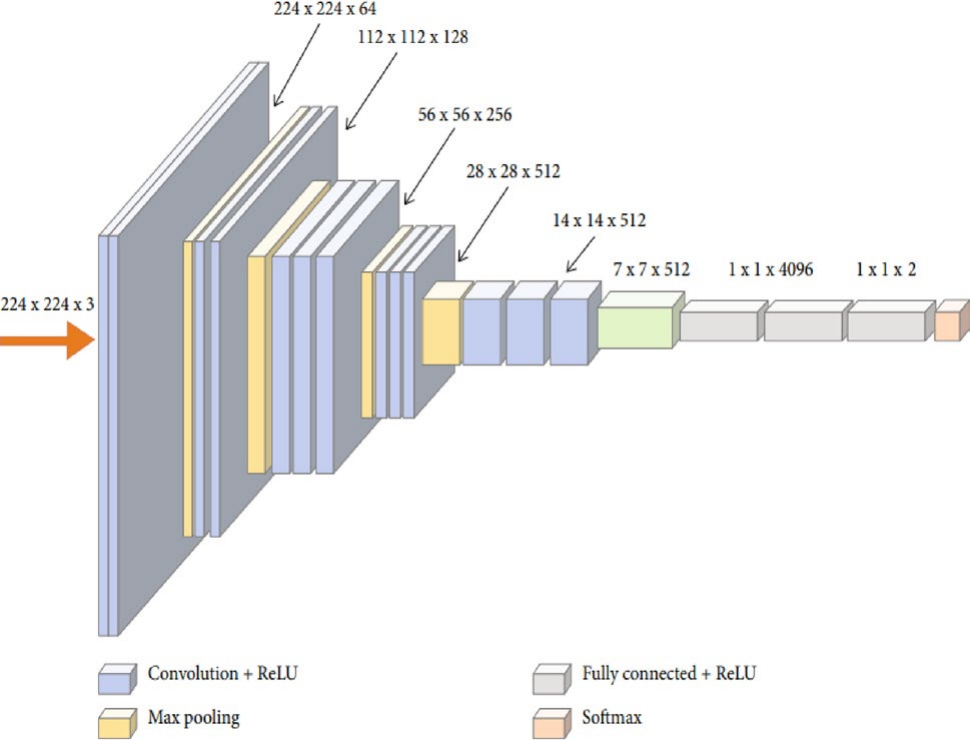
Modified version of the VGG16 model.

The original VGG16 model was initially trained to classify 1000 different object classes. However, the two classes of KUB x-ray images used in this study cannot be directly classified by the original VGG16 model. The current study introduces a model to classify KUB x-rays using a modified version of the VGG16 model (Fig. 4). This modified version of the VGG16 model enables the direct classification of the two KUB x-ray classes.

### XAI

According to^67^, explainability means the capacity to communicate how an AI decision has reached a broader range of end users in ways humans can comprehend. Many AI models, particularly those based on DL, have the potential to be challenging to understand. These models often involve millions of parameters and rely on complex patterns and correlations that are difficult to decipher. This complexity can raise concerns about bias, privacy, ethics, fairness, and transparency.

To address these concerns, XAI refers to the capability of AI systems to provide understandable and interpretable explanations for their decisions and actions, techniques that aim to enhance the comprehensibility and transparency of AI models. In this study, the LRP technique is used to determine which features of the DL model are responsible for specific predictions.

#### Layer-wise relevance propagation

For enhancing the explainability of networks utilizing the back-propagation algorithm, one of the principal algorithms employed is LRP^68^. A backward propagation technique called LRP gives relevance scores to a model's input features based on how much they contribute to the output. The most crucial neurons for the prediction are then identified through the model layers using the relevance scores. Additionally, LRP deals with the shortcomings of shattered gradients in gradient methods (Grad-CAM) and perturbation methods (occlusion maps)^69^.

## Simulation and results

We utilized Google Colab and PyTorch for simulation and obtaining results. Google Colab furnished the necessary computational resources, while PyTorch was an efficient framework for constructing and training DL models. Our performance assessment employed the metrics derived from Eqs. (1–8)^70,71^, wherein Kp/Sp represents true positives, Km/Sm denotes true negatives, Ke/Se signifies false positives, and Kn/Sn indicates false negatives. The computed metrics encompassed accuracy, misclassification rate, precision, sensitivity, specificity, FPR, FNR, and F1 Score.

*Accuracy* Accuracy is the proportion of correctly classified instances out of the total predictions made by a model, often represented as a percentage.

1 $${\text{Accuracy}} = \frac{{({\text{K}}_{{\text{p}}} /{\text{S}}_{{\text{p}}} ) + \left( {{\text{K}}_{{\text{m}}} /{\text{S}}_{{\text{m}}} } \right){ }}}{{({\text{K}}_{{\text{p}}} /{\text{S}}_{{\text{p}}} ) + \left( {{\text{K}}_{{\text{m}}} /{\text{S}}_{{\text{m}}} } \right){ } + ({\text{K}}_{{\text{e}}} /{\text{S}}_{{\text{e}}} ) + \left( {{\text{K}}_{{\text{n}}} /{\text{S}}_{{\text{n}}} } \right){ }}}{*}100$$

*Misclassification rate* The misclassification rate is the proportion of incorrectly classified instances out of the total predictions, usually expressed as a percentage or a fraction.

2 $${\text{Misclassification rate}} = \frac{{({\text{K}}_{{\text{e}}} /{\text{S}}_{{\text{e}}} ) + \left( {{\text{K}}_{{\text{n}}} /{\text{S}}_{{\text{n}}} } \right){ }}}{{({\text{K}}_{{\text{p}}} /{\text{S}}_{{\text{p}}} ) + \left( {{\text{K}}_{{\text{m}}} /{\text{S}}_{{\text{m}}} } \right){ } + ({\text{K}}_{{\text{e}}} /{\text{S}}_{{\text{e}}} ) + \left( {{\text{K}}_{{\text{n}}} /{\text{S}}_{{\text{n}}} } \right){ }}}{*}100$$

*Precision* Precision measures the ratio of true positive predictions to the total positive predictions made by a model, emphasizing the accuracy of positive classifications.

3 $${\text{Precision}} = \frac{{({\text{K}}_{{\text{p}}} /{\text{S}}_{{\text{p}}} ){ }}}{{({\text{K}}_{{\text{p}}} /{\text{S}}_{{\text{p}}} ) + \left( {{\text{K}}_{{\text{e}}} /{\text{S}}_{{\text{e}}} } \right){ }}}{*}100$$

*Sensitivity* Sensitivity calculates the proportion of true positive predictions relative to all actual positive instances, indicating a model's ability to identify positives correctly.

4 $${\text{Sensitivity}} = \frac{{({\text{K}}_{{\text{p}}} /{\text{S}}_{{\text{p}}} ){ }}}{{({\text{K}}_{{\text{p}}} /{\text{S}}_{{\text{p}}} ) + \left( {{\text{K}}_{{\text{n}}} /{\text{S}}_{{\text{n}}} } \right){ }}}{*}100$$

*Specificity* Specificity quantifies the ratio of true negative predictions to all actual negative instances, measuring a model's capacity to identify negatives correctly.

5 $${\text{Specificity}} = \frac{{\left( {{\text{K}}_{{\text{m}}} /{\text{S}}_{{\text{m}}} } \right)}}{{\left( {{\text{K}}_{{\text{m}}} /{\text{S}}_{{\text{m}}} } \right) + \left( {{\text{K}}_{{\text{e}}} /{\text{S}}_{{\text{e}}} } \right)}}{*}100$$

*FPR* FPR is the proportion of false positive predictions relative to all actual negative instances, demonstrating the model's tendency to misclassify negatives as positives.

6 $${\text{FPR}} = \frac{{\left( {{\text{K}}_{{\text{e}}} /{\text{S}}_{{\text{e}}} } \right)}}{{\left( {{\text{K}}_{{\text{e}}} /{\text{S}}_{{\text{e}}} } \right) + \left( {{\text{K}}_{{\text{m}}} /{\text{S}}_{{\text{m}}} } \right)}}{*}100$$

*FNR* FNR calculates the ratio of false negative predictions to all actual positive instances, illustrating the model's likelihood to misclassify positives as negatives.

7 $${\text{FNR}} = \frac{{\left( {{\text{K}}_{{\text{n}}} /{\text{S}}_{{\text{n}}} } \right)}}{{\left( {{\text{K}}_{{\text{n}}} /{\text{S}}_{{\text{n}}} } \right) + ({\text{K}}_{{\text{p}}} /{\text{S}}_{{\text{p}}} ){ }}}{*}100$$

*F1 Score* The F1 Score is the harmonic mean of precision and sensitivity, providing a single metric that balances both aspects of classification accuracy.

8 $${\text{F}}1{\text{ Score}} = \frac{{2{*}\left( {\text{Precision*Sensitivity}} \right)}}{{{\text{Precision}} + {\text{Sensitivity}}}}$$

For the model's training hyperparameters, we maintained the mini-batch size at 32, determined the optimal training epoch to be 10, applied a learning rate of 0.00001 during network training, and utilized the Adam optimization algorithm for the training process (Table 3 outlines each hyperparameter, accompanied by an explanatory note).

**Table 3:** Training hyperparameters.

Sr. no.	Parameters	Value	Explanatory note
1	Size of the Mini Batch	32	A mini-batch size of 32 processes 32 data samples together in each training iteration
2	No. of Epochs	10	It means training the modified VGG16 for 10 complete passes through the entire dataset
3	Learning Rate	0.00001	A learning rate 0.00001 signifies a small step size for updating model parameters during training
4	Optimization Algorithm	Adam	The optimization algorithm "Adam" combines adaptive learning rates and momentum to update model parameters during training efficiently

**Figure 5:**
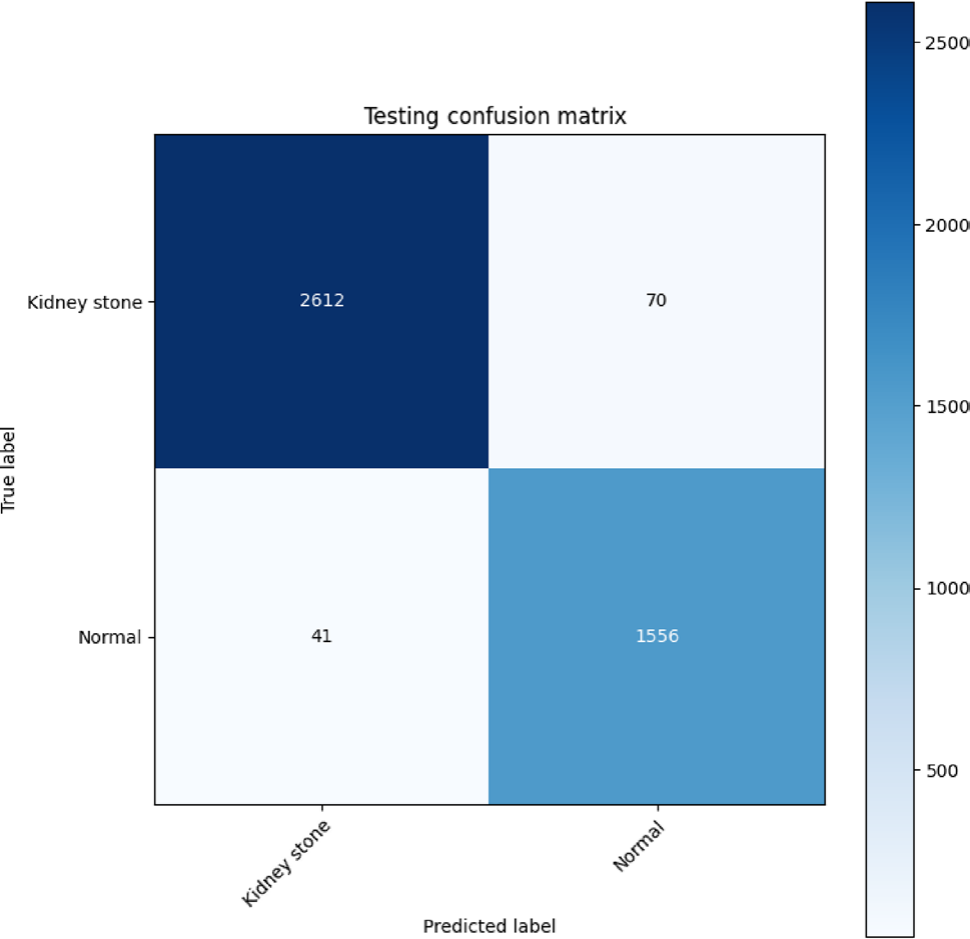
Testing confusion matrix for the modified VGG16 model.

Subsequently, we tested the modified VGG16 model to analyze a dataset comprising 4279 KUB X-rays, aiming to distinguish between X-rays featuring kidney stones and those categorized as normal (Fig. 5; Table 4). Regarding kidney stone X-rays from KUB, the model identified 2612 X-rays as kidney stones (true positives). While mistakenly labeling 70 X-rays as normal (false positives). For normal X-rays of KUB, the model correctly identified 1556 X-rays as normal (true negatives) and erroneously labeled 41 X-rays as kidney stones (false negatives).

**Table 4:** Statistical significance of each criterion for the modified VGG16 model.

Performance parameters	Testing
Accuracy	97.41%
Misclassification rate	2.59%
Precision	97.39%
Sensitivity	98.45%
Specificity	95.70%
FPR	4.30%
FNR	1.55%
F1 Score	0.98

Table 4 illustrates the statistical significance of each criterion for the modified version of the VGG16, including accuracy, misclassification rate, precision, sensitivity, specificity, FPR, FNR, and F1 Score.

**Figure 6:**
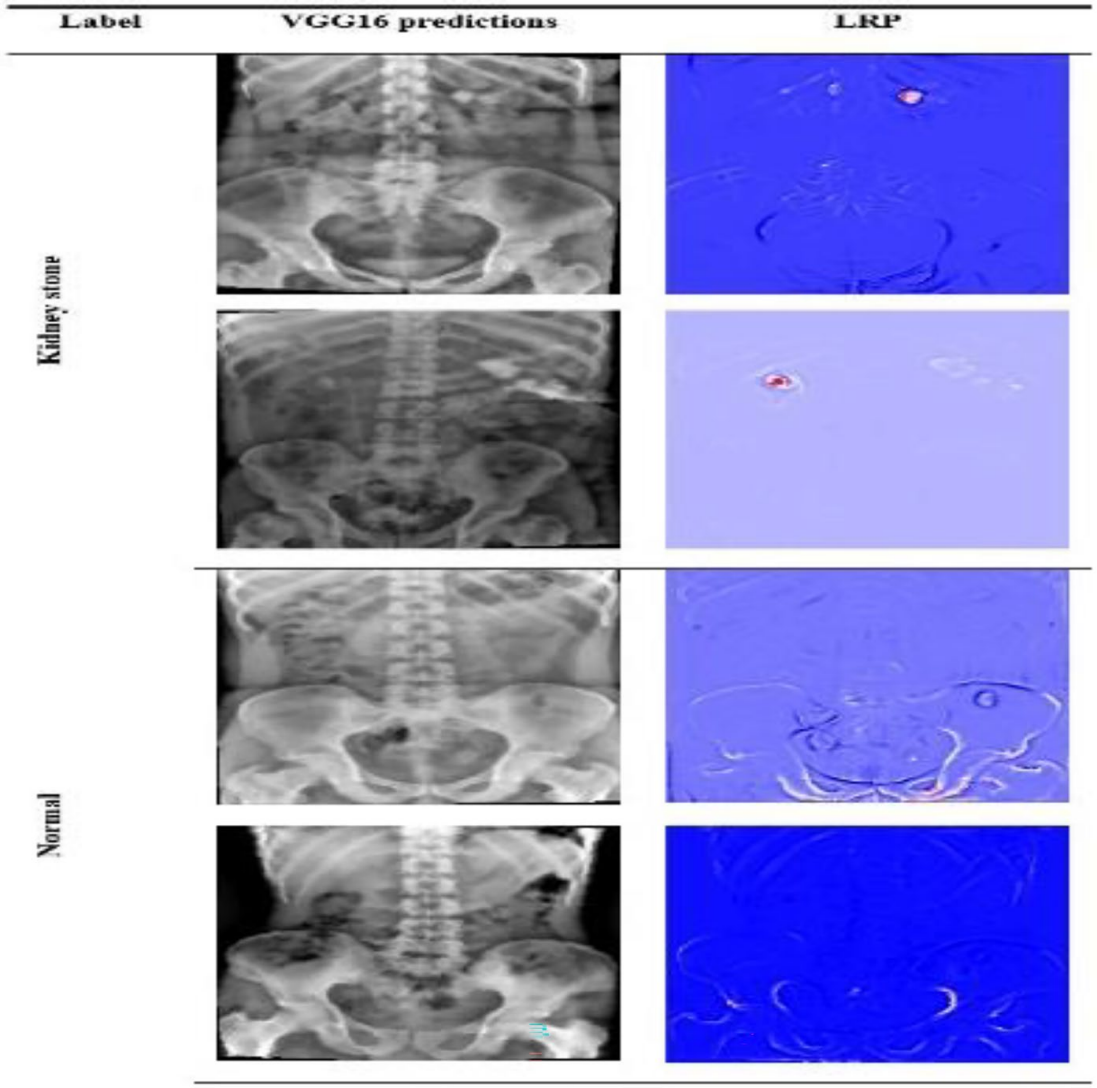
Explanations based on LRP for the modified VGG16 predictions.

Employing the LRP technique on the modified VGG16 model allowed us to pinpoint the regions in the KUB X-ray image that significantly contribute to the model's prediction of kidney stone presence. Notably, highlighted areas in KUB X-rays indicate the presence of kidney stones, while normal X-rays exhibit clarity and lack visible indications (Fig. 6).

Numerous ways have been utilized to detect kidney stones; nevertheless, TL is a revolutionary method for identifying the presence of kidney stones. Table 5 compares the proposed model's performance to previously reported state-of-the-art literature. The proposed model integrates modified VGG16 architecture with the XAI technique, significantly advancing kidney stone identification. This model distinguishes itself through exceptional performance, achieving a remarkable testing accuracy of 97.41% and an impressively low misclassification rate of 2.59%. Utilizing the XAI technique enhances the model's transparency and interpretability, addressing critical concerns related to the opacity of DL models. Additionally, the model benefits from a substantial dataset of 14,265 KUB x-ray images, enabling it to capture intricate patterns effectively.

**Table 5:** Comparison of the proposed model with state-of-the-art literature.

Author	Year	Dataset type	Technique	Use of XAI	Accuracy	Misclassification rate
Chiang et al.^47^	2003	105 healthy controls and 151 patients with calcium oxalate stones	ANN and DA	No	ANN: 89% and DA: 75%	ANN: 11% and DA: 25%
Dussol et al.^48^	2006	119 stone formers and 96 controls	ANN (LDA and MVDA)	No	LDA: 75.8% and MVDA: 74.4%	LDA: 24.2% and MVDA: 25.6%
Cauderella et al.^49^	2011	80 patient’s data	ANN and LR	No	ANN: 88.8% and LR: 67.5%	ANN: 11.2% and LR: 32.5%
Kumar and Abhishek^50^	2012	Data from 1000 patients	LVQ, MLP, and RBF	No	LVQ: 84%, MLP: 92%, and RBF: 87%	LVQ: 16%, MLP: 8% and RBF: 13%
Ebrahimi and Mariano^51^	2015	KUB CT scan slides from 39 patients	Image processing techniques and geometry principles	No	84.61%	15.39%
Kazemi and Mirroshandel^52^	2018	Numeric characteristics from 936 patients	Ensemble learning model	No	97.1%	2.9%
Li and Elliot^53^	2019	1874 CT KUB reports	NLP	No	85%	15%
De Perrot et al.^54^	2019	416 patient data	ML model	No	85.1%	14.9%
Kahani et al.^55^	2020	KUB x-ray images	LASSO with ML classifiers	No	96%	4%
Jungmann et al.^56^	2020	1714 LDCT images	NLP	No	72%	28%
Annameti Rohith et al.^57^	2021	114 ultrasound images	Median and rank filters	No	Median filter: 86.4%, and rank filter: 82.2%	Median filter: 13.6%, and rank filter: 17.8%
Suresh and Abhishek^58^	2021	KUB ultrasound images	Image processing techniques	No	92.57%	7.43%
Jendenber et al.^59^	2021	NCCT images of 341 patients containing a distal ureteral stone, phlebolith, or both	CNN	No	92%	8%
Cui et al.^60^	2021	625 CT images	DL and threshold-based model	No	90.30%	9.70%
Yildirim et al.^61^	2021	1799 coronal CT images	XResNet-50	No	96.82%	3.18%
Tsitsiflis et al.^62^	2022	Medical data of 716 patients	ANN	No	81.43%	18.57%
Valencia et al.^63^	2022	CT scans of around 40 patients	Image processing	No	92.5%	7.5%
Proposed model	2024	14,265 KUB x-ray images	VGG16 empowered with LRP	Yes	97.41%	2.59%

## Conclusion

Kidney stone formation can lead to a significant obstruction in renal function, consequently affecting human health and survival. As a result, the prompt identification and prediction of kidney stones assume critical importance. Recent technological advancements have enabled the broad integration of ML and DL methodologies into diagnosing kidney stones. In this study, we introduced and used a modified VGG16 model to identify kidney stones in KUB x-ray images. The results of our experiments show that the modified VGG16 model has an accuracy of 97.41% in identifying kidney stones within KUB x-ray images.

DL models like VGG16 can be perceived as “black boxes” because they lack transparency or prediction fairness. In addressing this issue, the study employs the XAI technique LRP to elucidate the model's predictions, enhancing users’ comprehension of the rationale behind the decision-making process. This approach provides a transparent and effective solution for arriving at definitive diagnostic conclusions, reducing the time needed for diagnosis and enhancing diagnostic accuracy.

## Limitations and future work

One of the critical limitations of our research is the availability of high-quality and diverse medical image data of KUB X-rays of kidney stones. The quality and diversity of the dataset are crucial in identifying kidney stones. In the future, overcoming this limitation will require continued efforts to collect, curate, and make a broader range of medical image data more readily available to improve model performance.

Even using XAI techniques such as LRP, the model’s interpretation may still be inconspicuous or might not give meaningful insight into the model's decision-making. In the future, further research in advanced XAI techniques and methodologies will have the potential to visually enhance the transparency, fairness, and interpretability of the model’s predictions, allowing users to understand better and trust the model.

The development of AI-based medical diagnosis enables personalized and science-based approaches to medical care. However, ethical considerations must be carefully weighed; strategies must be developed to mitigate patient privacy data security and algorithmic bias and to minimize unintended consequences of AI-based medical diagnosis. Blockchain technology can address patient privacy and data security in future work by providing decentralized storage and secure access controls for patient data. With blockchain, the training of the AI models is transparent and auditable, improving algorithmic bias and enabling accountability, which is a cornerstone of trusted AI-based medical diagnosis.

The current study focused on developing and evaluating the proposed model. In the future, the proposed model's computational complexity and resource requirements will be analyzed to determine its size.

## Data Availability

The dataset & Simulation files used during the current study are available from the corresponding author upon reasonable request.

## Code availability

Current code version: V1.0. Permanent link to code/repository used for this code version: https://github.com/deepfindr/xai-series/blob/master/05_lrp.py. Software code languages, tools and services used: Python 3 (Used in Google Colab). Compilation requirements, operating environments, and dependencies: CUDA GPU, Microsoft Windows. Support email for questions: ara4013@qatar-med.cornell.edu , adnan@gachon.ac.kr.
