# Author Correction: Identification of kidney stones in KUB X-ray images using VGG16 empowered with explainable artificial intelligence

**DOI:** 10.1038/s41598-024-60554-0

**Published:** 2024-05-06

**Authors:** Fahad Ahmed, Sagheer Abbas, Atifa Athar, Tariq Shahzad, Wasim Ahmad Khan, Meshal Alharbi, Muhammad Adnan Khan, Arfan Ahmed

**Affiliations:** 1 School of Computer Science, National College of Business Administration and Economics, Lahore, 54000 Pakistan; 2 Department of Computer Sciences, Bahria University, Lahore Campus, Lahore, 54000 Pakistan; 3 Department of Computer Science, Comsats University Islamabad, Lahore Campus, Lahore, 54000 Pakistan; 4 Department of Computer Sciences, COMSATS University Islamabad, Sahiwal Campus, Sahiwal, 57000 Pakistan; 5 Department of Computer Science, College of Computer Engineering and Sciences, Prince Sattam Bin Abdulaziz University, 11942 Alkharj, Saudi Arabia; 6 School of Computing, Skyline University College, University City Sharjah, 1797 Sharjah, UAE; 7 Department of Software, Faculty of Artificial Intelligence and Software, Gachon University, Seongnam-si, 13120 Republic of Korea; 8 Riphah School of Computing and Innovation, Faculty of Computing, Riphah International University, Lahore Campus, Lahore, 54000 Pakistan; 9 AI Center for Precision Health, Weill Cornell Medicine-Qatar, Doha, Qatar

Correction to: *Scientific Reports* 10.1038/s41598-024-56478-4, published online 14 March 2024

The original version of this Article contained an error in the Funding section.

"This research work is supported by Qatar National Library."

now reads:

"The publication of this article was funded by the Weill Cornell Medicine – Qatar Health Sciences Library."

The original Article has been corrected.

